# Efficacy of Type 2 PRRSV vaccine against challenge with the Chinese lineage 1 (NADC30-like) PRRSVs in pigs

**DOI:** 10.1038/s41598-019-47239-9

**Published:** 2019-07-25

**Authors:** Chunhua Wei, Ailing Dai, Jialin Fan, Yan Li, Anni Chen, Xia Zhou, Manlin Luo, Xiaoyan Yang, Jiankui Liu

**Affiliations:** 1 College of Life Sciences of Longyan University, Longyan, 364012 Fujian China; 2 Fujian Provincial Key Laboratory for the Prevention and Control of Animal Infectious Diseases and Biotechnology, Longyan University, Longyan, 364012 Fujian China; 3 College of Veterinary Medicine, South China Agricultural University, Guangzhou, 510642 Guangdong China

**Keywords:** Live attenuated vaccines, Genetic variation

## Abstract

The objective of the present study was to determine the cross-protection of Ingelvac PRRS MLV against challenge with the new lineage 1 PRRSV emerged in China in pigs. Two lineage 1 PRRSV strains (FJZ03 and FJWQ16 originated from recombination event between NADC30 and JXA1-like strain). We found that pigs vaccinated with the vaccine were protected against challenge with the FJZ03 as shown by fewer days of clinical fever, reduced lung pathology scores, lower PRRS virus load in the blood and developed broadly neutralizing antibodies with high titers to FJZ03. In contrast, vaccine provided limited protection against challenge with FJWQ16 with higher fever, lower antibody titers, lower neutralizing antibodies and higher viral loads in blood. These results demonstrate PRRSV-MLV provides incomplete protection against new lineage 1 PRRSVs.

## Introduction

Porcine reproductive and respiratory syndrome (PRRS) is a global viral swine disease and characterized by severe reproductive failure in sows and respiratory problems in piglets^1^. PRRS virus (PRRSV) is a small, enveloped, positive-sense single-stranded RNA virus belonging to the *Arteriviridae* family in the *Nidovirale*s order^2–4^. The PRRSV genome is about 15 kb in length and codes for at least 16 non-structural proteins (nsps) (nsp1α, nsp1β, nsp2-6, nsp2TF, nsp2N, nsp7a, nsp7b and nsp8-12) and 8 structural proteins (GP2, E, GP5a, GP3-GP5, M and N)^4–9^.

PRRSV strains were divided into two distinct genotypes, type 1 (European strains, EU) and type 2 (North American strains, NA), which share only about 60% nucleotide identity at the genome level with distinct antigenicity. PRRSV emerged in China in 1996, and has become one of the country’s most serious swine diseases. In May 2006, highly pathogenic PRRS (HP-PRRSV) emerged in China and is characterized by high fever and associated with a high mortality rate, resulting in immense economic losses in the swine industry in China^10^. In 2013, new lineage 1 PRRSV (NADC30-like) emerged in China and spread widely in Chinese swine herds with high morbidity and mortality in sows and piglets. The NADC30-like PRRSV is now the predominant strain circulating in swineherds and have been causing great economic loss in China. Vaccination is the most effective and practical method to prevent infectious diseases and live attenuated PRRSV vaccines have been a valuable tool in PRRS disease control^11^. To date, several live attenuated NA-type PRRSV vaccines, such as Ingelvac PRRS^®^ MLV, CH-1R, R98 and HP-PRRSV-derived live vaccines including JXA1-R, HuN4-F112 and TJM are commercially available in China. Among these vaccines, Ingelvac PRRS MLV (MLV) is one of the most widely used in China, and MLV strain belong to lineage 5 virus, whereas NADC30-like PRRSV is lineage 1 according to the global PRRSV classification systems^12^. Recently, some novel recombinant PRRSV strains (lineage 1) arising from NADC30-like PRRSV and filed or vaccine strain in China have been reported, whether there is cross-protection from the commercial vaccine against the novel recombinant PRRSV and NADC30-like PRRSV challenge is not known. Therefore, the objective of this study was to evaluate the efficacy of PRRSV vaccine MLV against heterologous NADC30-like PRRSV challenge in pigs.

## Results

### Complete genomic sequence analysis

Excluding poly(A) tails, complete genome sequence of FJZ03 and FJWQ16 strains was 15,016 nucleotides in length. Genome alignments revealed 81.8–96.0% identity with NA-type PRRSV strains, and identity was 92.0% between the two strains. Of note, FJZ03 and FJWQ16 had the maximum identity (97.1% and 93.0%, respectively) with NADC30. Furthermore, strains FJZ03 and FJWQ16 share 86.0–86.2% homology with the genome of vaccine strain Ingelvac PRRS MLV (MLV), however, 83.9–85.2% homology with the genome of JXA1-R vaccine (a live attenuated virus vaccine strain derived from the highly pathogenic PRRSV JXA1).

**Figure 1:**
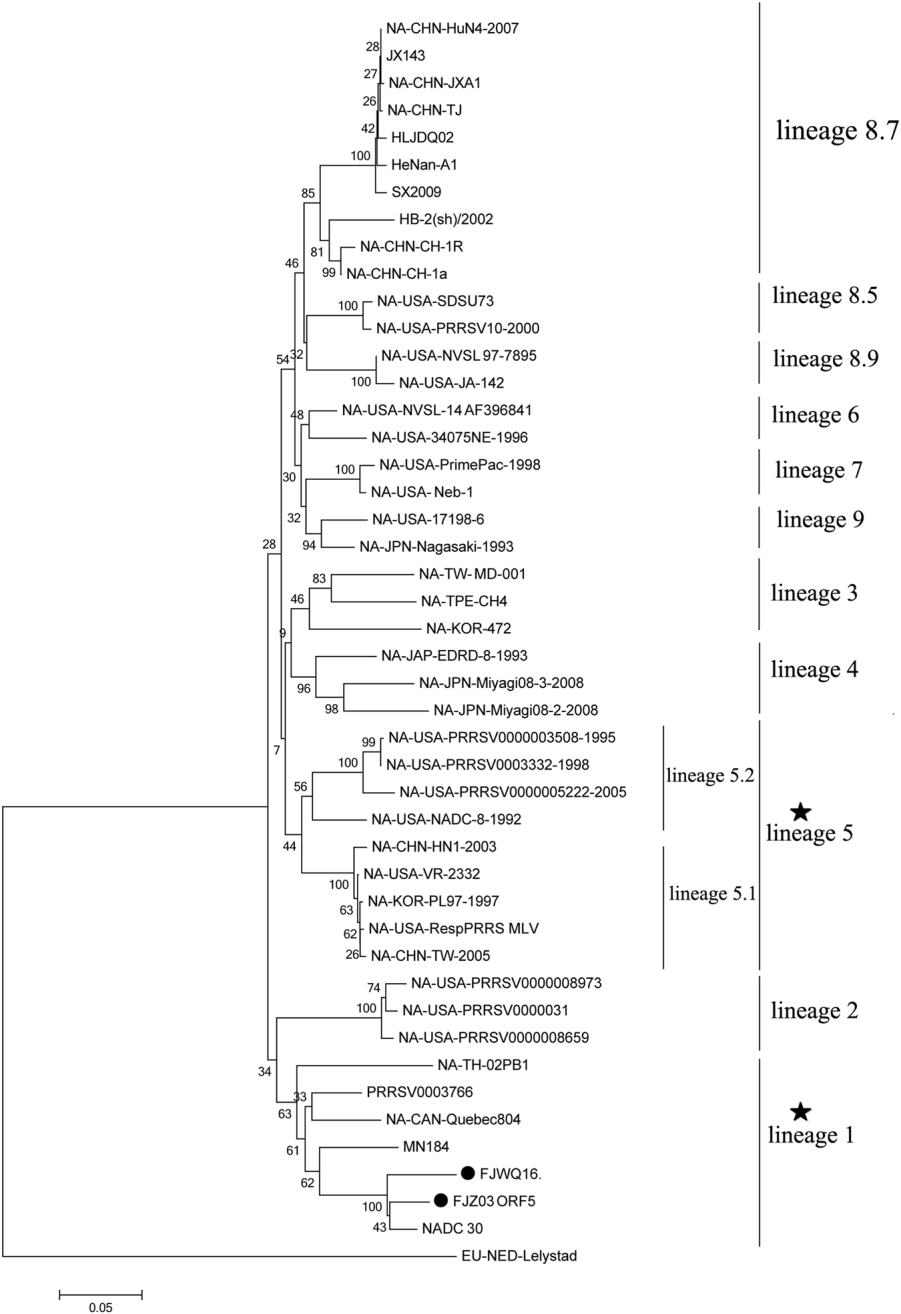
Phylogenetic tree based on the ORF5 genes of the 2 isolates and reference viruses. Reliability of the tree was assessed by bootstrap analysis of 1000 replications. Our representative isolates were marked with the black cicrle (•).

**Figure 2:**
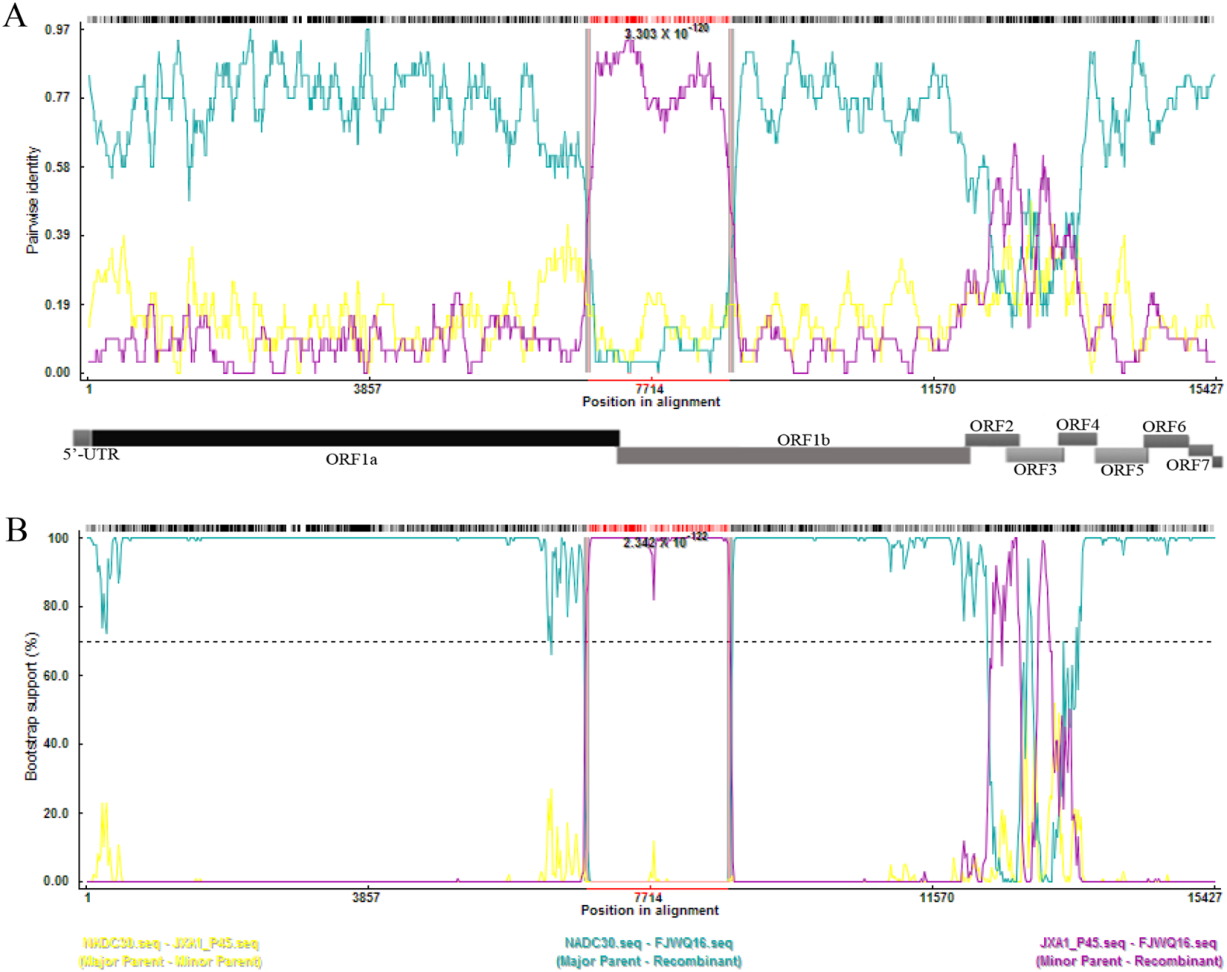
Recombination between the JXA1 P45 and NADC30 was detected in FJWQ16 by means of RDP (**A**) and boot scanning. (**B**) One recombination break points were detected at positions 6854–8787 of the sequence alignment (with reference to VR-2332 strain).

High genetic diversity is a significant characteristic of PPRSV. A systematic classification of type 2 PRRSV has been conducted based on analysis of 8624 ORF5 sequences in the databases including field isolates and vaccine strains^12^. In this system, type 2 PRRSV was divided into nine monophyletic lineages (1–9). Therefore, this type of systematic genotyping was used for PRRSV classification in the present study.The phylogenetic analysis of ORF5 genes showed that FJZ03 and FJWQ16 formed a minor branch represented by NADC30 (lineage 1 virus) (Fig. 1). To confirm the potential recombination event, we evaluated potential recombinants using seven algorithms (RDP, GeneConv, BootScan, MaxChi, Chimera, SiScan, and 3Seq) implemented in RDP 4.80. Two inter-lineage recombination events between lineages 1 (NADC30) and 8.7 (JXA1 P45) were identified in the FJWQ16, with recombination breakpoints at position 6854–8787 (located in nsp7 (nt62)-nsp9 (nt1103), with reference to VR-2332 strain, Fig. 2). However, we note that we failed to observe any evidence of recombination in the FJZ03 by using RDP software.

### Clinical observations

**Figure 3:**
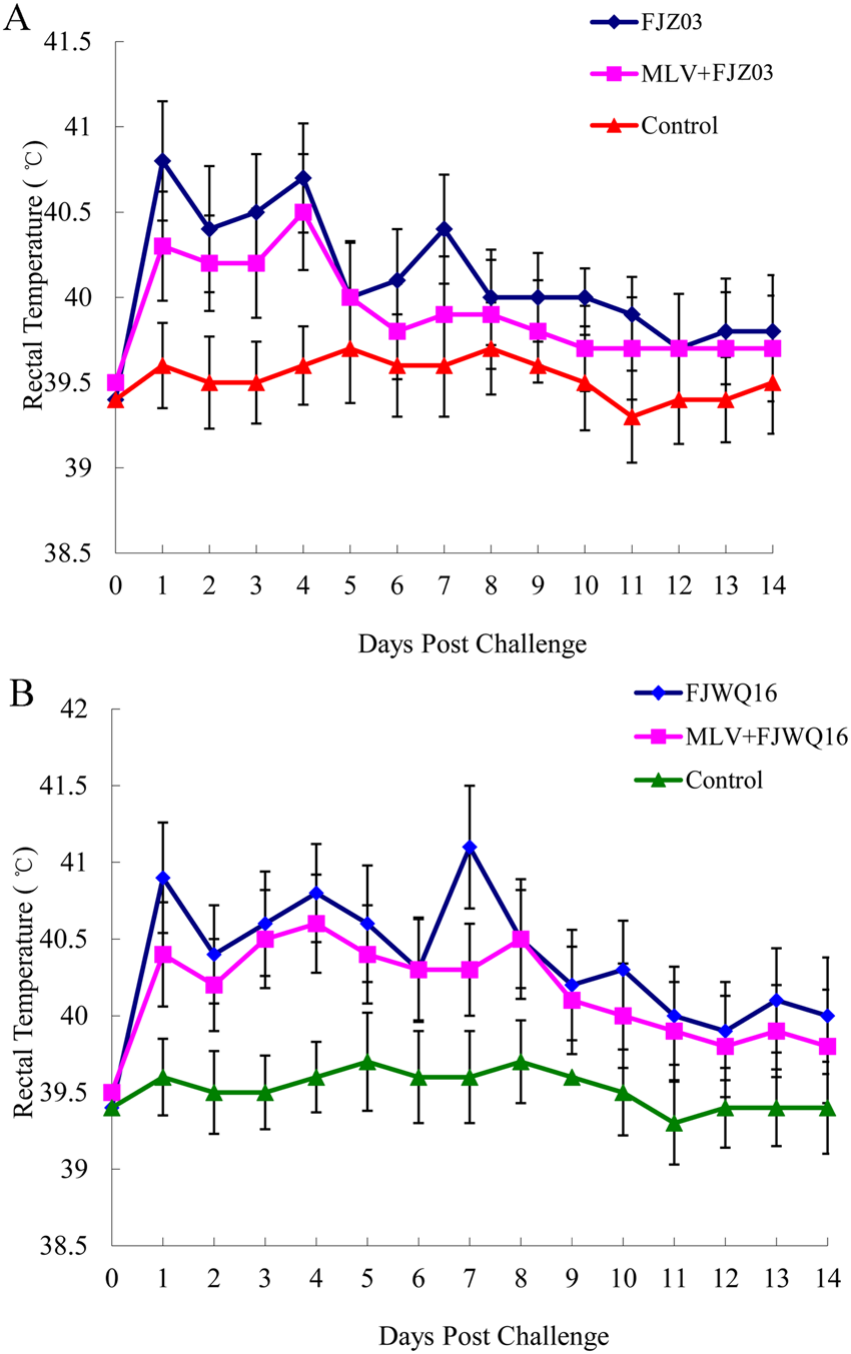
Comparison of mean rectal temperatures (±S.D.) among vaccinated, non-vaccinated and control groups: (**A**) FJZ03 challenge groups; (**B**) FJWQ16 challenge groups. The data were expressed as the mean ± S.D. of the numbers of pigs alive at the time of the sample collection.

**Figure 4:**
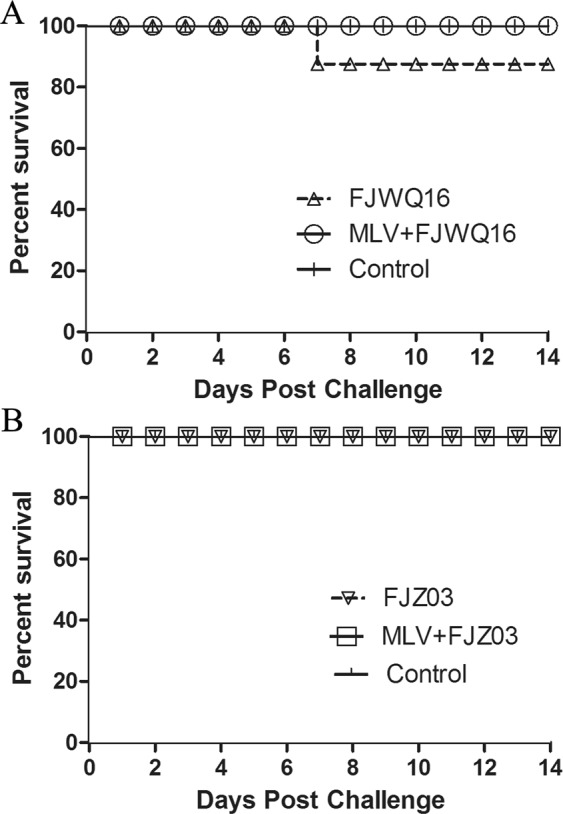
Survival rates of piglets after challenge with FJWQ16 (**A**) or FJZ03 (**B**).

**Figure 5:**
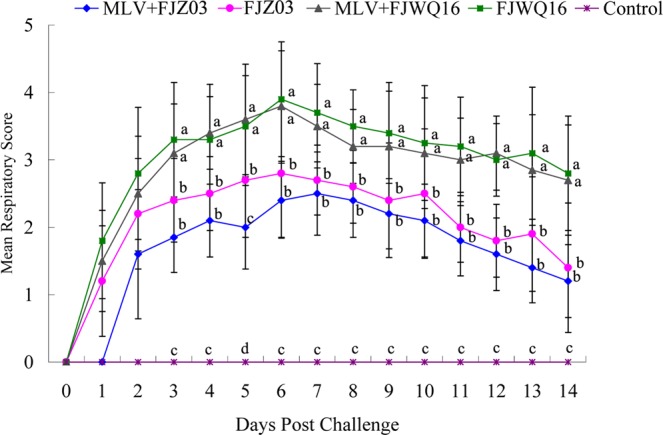
Mean respiratory score from pigs in vaccinated, non-vaccinated and control groups. Different letter superscripts denote significant differences at *p* < 0.05.

**Figure 6:**
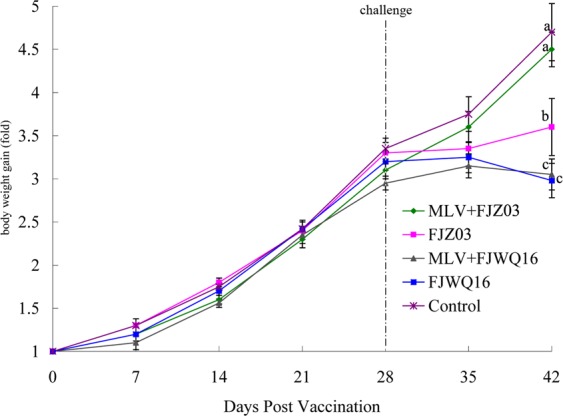
Fold of total body weight gain among vaccinated, non-vaccinated and control groups was calculated by considering the weight of the pig on day 0 as 1. Different letter superscripts denote significant differences at *p* < 0.05.

Differences in clinical expression between group 1 (MLV + FJZ03) and group 2 (MLV + FJWQ16) pigs were observed. The mean rectal temperatures was high (>40 °C) in the MLV + FJZ03 group from 1 to 4 dpc, then leveled off (Fig. 3A), whereas mean rectal temperatures was above 40 °C from 1 to 9 dpc in the MLV + FJWQ16 group 2 (Fig. 3B). All pigs in MLV + FJWQ16 group exhibited severe clinical signs including high fever, acute respiratory distress and coughing during 2–10 dpc, whereas pigs from the MLV + FJZ03 group showed no significant clinical signs throughout the experiment. The mean rectal temperature in pigs unvaccinated and challenged with FJWQ16 and those vaccinated and challenged with FJWQ16 were significantly higher (*p* < 0.05) than in pigs vaccinated and challenged with FJZ03 and control group at 5 dpc, 7 dpc, 8 dpc and 10 dpc (Fig. 3). Unvaccinated pigs challenged with FJZ03 or FJWQ16 developed high fever (≥40 °C) from 1 to 10 dpc and typical symptoms of PRRS such as acute respiratory distress, lethargy, anorexia, and emaciation from 2 to 10 dpc. Surprisingly, there were not statistical significant differences in rectal temperatures (except 7 dpc) between MLV + FJWQ16 and unvaccinated + FJWQ16 challenge group (Fig. 3B). Survival rates of pig in unvaccinated by challenged with FJWQ16 group was 80%, while the pigs in unvaccinated by challenged with FJZ03 group was 100% (Fig. 4). The mean respiratory scores were significantly higher (*p* < 0.05) in pigs unvaccinated challenged pigs and vaccinated challenged with FJWQ16 with or without vaccination pigs than pigs in other 2 groups from 3 to 14 dpc (Fig. 5). The body weight gain of pigs was monitored throughout the study. As shown in Fig. 5, by the end of the first week after challenge (on 7 dpc), pigs in unvaccinated + FJWQ16, unvaccinated + FJZ03 and MLV + FJWQ16 groups showed low weight gain, whereas in both unvaccinated + FJWQ16 and MLV + FJWQ16 groups lost weight during 7–14 dpc. Control pigs gained weight over the duration of the study. The pigs in the unvaccinated + FJWQ16 and MLV + FJWQ16 challenge groups had significantly (*p* < 0.05) lower body weight gain than that of MLV + FJZ03 group and control group during 7–14 dpc (Fig. 6).

### Lung pathology

Macroscopic lung lesions were characterized by symptoms of interstitial pneumonia, multifocal, scattered bleeding points, tan-mottled areas with irregular and indistinct borders. The scores for gross lung lesion were shown in Table 1. In two unvaccinated groups, pigs infected with FJWQ16 showed more severe pathology than FJZ03 infected pigs (*p* < 0.05). Gross lung lesions in the MLV + FJWQ16 group were significantly more severe than those in MLV + FJZ03 and control groups (*p* < 0.05). However, no pathologic lesions were observed in control pigs. Microscopic lung lesions in unvaccinated + FJWQ16, unvaccinated + FJZ03, MLV + FJWQ16-infected pigs were characterized by interstitial pneumonia with severely alveolar septa thickening, increased numbers of lymphocytes and interstitial macrophages. Mild microscopic lung lesions were observed in MLV + FJWQ16-infected and MLV + FJZ03-infected pigs, whereas no noticeable microscopic lung lesions were observed in the control pigs.

**Table 1:** Macroscopic scores of lung after infection.

Designation	Number	Macroscopic score (lung)			
		Mean ± S.D.	Pathological changes		
			*	**	***
FJZ03	5	39.2 ± 14.7	1	3	1
MLV + FJZ03	5	29.8 ± 10.3	3	2	0
FJWQ16	5	57.8 ± 18.1	0	1	4
MLV + FJWQ16	5	51.8 ± 13.8	0	2	3
Control	5	12 ± 3.7	5	0	0

*Macroscopic scores ≤ 30; **Macroscopic scores between 30 and 50; ***Macroscopic scores ≥ 50.

### Detection of virus in the serum

**Figure 7:**
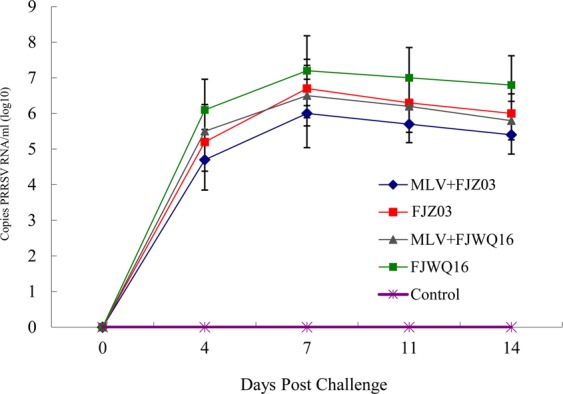
Mean values of the genomic copy number of PRRSV RNA in serum samples. The data were expressed as the mean ± S.D. of the numbers of pigs alive at the time of the sample collection.

Genomic copies of the vaccine virus were detected in the sera from the vaccinated and challenged pigs at 4 dpi, and had a low serum viral load 28 days post immunization. In the pigs from the MLV + FJZ03 group, high level of the mean serum viral load was detected (10^4.7^ copies/mL) on 4 dpc, which peaked at 10^6.0^ copies/mL on 7 dpc, and then decreased (Fig. 7). After challenge with FJWQ16, the pigs in the MLV + FJWQ16 group had a high mean serum viral titer on 4 dpc (10^5.5^ copies/mL), which peaked (10^6.5^ copies/mL) on 7 dpc and remained high until 14 dpc (Fig. 7). In the unvaccinated + FJZ03 and unvaccinated + FJWQ16 groups, viral load was detected starting on 4 dpc (10^5.2^ copies/mL, 10^6.1^ copies/mL, respectively), which peaked on 7 dpc (10^6.7^ copies/mL, 10^7.2^ copies/mL, respectively) and remained high until 14 dpc (Fig. 7). Vaccinated and challenged pigs had a significantly (*p* < 0.05) lower number of genomic copies of type 2 PRRSV in the blood than the unvaccinated and challenged pigs from 7 to 11 dpc (Fig. 7).

### Anti-PRRSV IgG antibodies

In all unvaccinated pigs, mean S/P ratio for every pig was 0.0 at 0 dpc. After FJZ03 and FJWQ16 challenge, all serum samples from unvaccinated + FJZ03 and unvaccinated + FJWQ16 group became PRRSV-positive with S/P ratio ≥ 0.4 occurred at 7 dpc, and by 14 dpc all pigs developed antibodies with a mean S/P ratio for the FJZ03 and FJWQ16 groups of 1.23 ± 0.12, and 1.48 ± 0.14, respectively. In all vaccinated pigs, seroconversion occurred on 14 dpi and the mean S/P ratio was 0.86 ± 0.15 for the MLV + FJZ03 group at 0 dpc, and 1.63 ± 0.18 at 14 dpc after FJZ03 challenge. At 0 dpc, the mean S/P ratio was 0.90 ± 0.15 for the MLV + FJWQ16 group, 1.78 ± 0.16 at 14 dpc after FJWQ16 challenge. Anti-PRRSV antibody titers were not detected in the negative control group throughout the experiment.

### PRRSV-specific neutralizing antibodies

**Figure 8:**
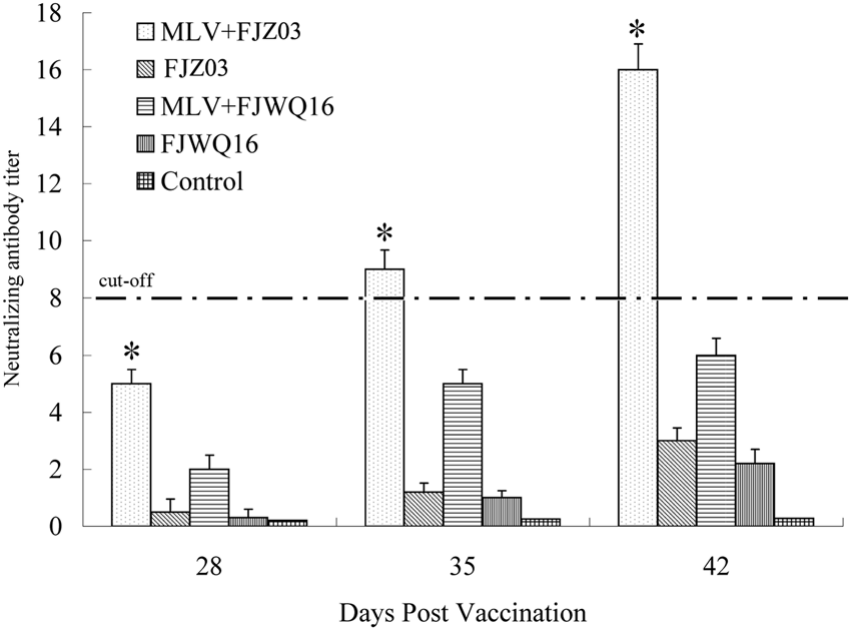
Serum neutralizing antibodies were detected using PRRSV infection of Marc-145 cells. Asterisk denotes that the NA titer of MLV + FJZ03 challenge group had a significantly higher than other groups (*p* < 0.05). The error bars represent standard deviations of the three experiments.

Neutralizing antibodies (NA) were detected in pigs vaccinated with MLV on 28 dpi. Pigs vaccinated with MLV developed high NA titers for FJZ03 (>4, Fig. 8), but only low titers for FJWQ16 (≤2, Fig. 8). Furthermore, the NA titers to FJZ03 increased in vaccinated pigs following challenge (>8), but the titers to FJWQ16 maintained at a low level (≤6) and never reached protective levels (Fig. 8). NAs were not detected in any pigs in the control group throughout the experiment.

## Discussion

PRRS has been causing substantial economic losses in the swine industry, especially, the HP-PRRSV, since 2006, in China^10^. Recently, new lineage 1 PRRSV (NADC30-like) emerged in China and is now a severe threat to Chinese pig population. The PRRSV-MLV vaccine can induce a protective immune response, but it may not have broad cross-protection against all isolates^13–16^. Although six PRRSV vaccines are currently marketed in China, Ingelvac PRRS MLV is one of the most widely used in China. In the present study, FJWQ16 and FJZ03 are lineage 1 virus (NADC30-lile PRRSV), while vaccine strain MLV is lineage 5 based on ORF5 analysis. However, whether there is cross-protection from the vaccine MLV (lineage 5) against heterologous NADC30-like PRRSV (lineage 1) challenge is not known. Therefore, the aim of the present study was to investigate the efficacy of Ingelvac PRRS MLV in a challenge study with the NADC30-like PRRSVs.

Ingelvac PRRS MLV has been shown to provide complete protection from infection by VR-2332, however, offers incomplete protection against newly emerging heterologous strains^13,14^. There is relatively little data pertaining to the immune responses and efficacy of MLV vaccine against challenges with NADC30-like PRRSV strains. In the present study, we found different levels of protection against challenge with NADC30-like PRRSVs following vaccination with the MLV. PRRSV viremia plays a key factor in the development of respiratory diseases^16^. The efficacy of vaccine is usually assessed by the reduction in viral loads after challenge with a virulent PRRSV^17–19^. Hence, the reduction of PRRSV viral loads in the blood and lungs is an important parameter to determine the efficacy of PRRSV vaccines^17–19^. In the present study, vaccine reduced PRRSV viral loads significantly in vaccinated and challenged groups compared with unvaccinated and challenged groups, however, in the vaccinated and challenged group (MLV + FJWQ16), the viral load remained high from 4 to 14 dpc. The high levels PRRSV viremia in MLV + FJWQ16 group result in significant respiratory diseases. Severity of the interstitial pneumonia is correlated with the amount of viral load^20^, in addition, PRRSV virulence was associated with higher titers of virus *in vivo*^21^. MLV + FJWQ16 group had high overall viral serum titers at 8 days after challenge, vaccination of pigs with MLV reduced only partially the severity of FJWQ16-induced lung lesions and the amount of PRRSV antigen within the interstitial pneumonia. Overall, compared to control and MLV + FJZ03 group, the MLV + FJWQ16 group had more days with high fever, exhibiting higher levels of viremia and significant body weight lost after FJWQ16 challenge, indicating that pigs vaccinated with MLV were not protected from FJWQ16 challenge. Although the use of PRRSV-MLV reduced body temperatures and viral load in the present study, there was a significant difference between MLV + FJZ03 and MLV + FJWQ16 groups. We have no clear explanation why MLV has different results of protection against FJZ03 and FJWQ16 challenge. It may be attributed to antigenic differences between the vaccine and the challenge strains, that is, MLV vaccine is less effective in inducing cross-protection against viral isolates that are significantly different from the vaccine strains. These results in present study agree with previous findings that commercial modified live PRRS vaccine is partially protect heterologous PRRS challenge^13,14,22–24^. It may be due to remarkable antigenic variation between the vaccine strains and challenging PRRSV strains^25^.

Neutralizing antibodies might play a significant role in protection against PRRSV^17,26^. In this study, neutralizing antibody titers to FJZ03 were present at protective titers (>8) in the vaccinated pigs after challenge with FJZ03. However, the titers to FJWQ16-specific neutralizing antibodies in pigs vaccinated with MLV never reached protective levels, indicating that vaccination with MLV does not induce broadly neutralizing antibodies to FJWQ16.

Although commercial vaccines provide limited protection to NADC30-like PRRSV infection, previous studies have focused on only one NADC30-like PRRSV strain^24,27,28^. The aim of the present study was to investigate the efficacy of the commercially available PRRSV type 2 vaccine MLV in a challenge study with two different NADC30-like PRRSV isolates. Phylogenetic and molecular evolutionary analyses indicated that FJWQ16 originated from recombination event between NADC30 and JXA1-like PRRSV strain (JXA1 P45), whereas FJZ03 was not observed any evidence of recombination between PRRSV. Additionally, recombinant PRRSVs (JL580 and FJ1402) between NADC30-like PRRSV and HP-PRRSV were shown to have higher pathogenicity than NADC30-like PRRSV. In the present study, recombinant strain FJWQ16 has higher pathogenicity than FJZ03 and agree with previous studies^28,29^. Furthermore, the JXA1-R vaccine virus was shown to have reverted to virulence under field conditions^30^. This JXA1 P45 strain maybe related to the virulence reversion of the JXA1-R vaccine virus circulating in swineherds. Thus, it is critical to monitor of PRRSV evolution in China. FJWQ16 replicated in serum at a higher titer than did FJZ03, and one piglet in the FJWQ16 group died, whereas the piglets in the FJZ03 group and control group survived throughout the experiment. Recently, the recombination between NADC30-like PRRSV and Chinese field strains/vaccine strains have been reported in many studies; these recombinant viruses showed more virulent than NADC30-like PRRSV, such as strain JL580, 14LY01-FJ, 14LY02-FJ 15LY01-FJ, and 15LY02-FJ from the recombination between NADC30 and HP-PRRSVs, which has been confirmed as highly pathogenic. In the present study, a recombinant type 2 PRRSV FJWQ16 between NADC30-like PRRSV and JXA1-like PRRSV was shown to have higher pathogenicity (20% mortality rate) than FJZ03, NADC30, and NADC30-like strain^28,29,31^. These results showed that genomic recombination is an important mechanism that plays a role in conditioning pathogenicity and antigenicity of the virus.

In summary, although a commercial MLV vaccine could shorten the period of fever, reduce levels of vermeil in sera and mortality rate, it was not able to reduce the clinical symptoms and lung lesions of the challenged pigs. These findings indicate that MLV vaccine provided incomplete cross-protection against newly emerging heterologous NADC30-like PRRSV and recombinant PRRSV infection. It is thus urgent to develop novel, safe and effective vaccines for controlling NADC30-like PRRSV.

## Materials and Methods

### Animal ethics

The animal study and all pig experimental procedures were performed in accordance with the guidelines of South China Agricultural University Institutional Animal Care and Use Committee (SCAU-AEC-2014-10) and approved by animal Ethics committee of South China Agricultural University.

### Virus and vaccine

Type 2 PRRSV FJZ03 strain (GenBank ID: KP860909) was isolated from lung samples of an aborted fetus in 2013 and FJWQ16 strain (GenBank ID: KX758249) from a herd with high morbidity and mortality in sows and piglets respiratory disease, in 2015. PRRSV was isolated from the positive sera and tissues using MARC-145 cells and real-time RT-PCR analysis was applied on the supernatants to confirm the growth of the virus. The virus-infected cells in 6-well plates were determined by indirect fluorescent antibody assay (IFA). The cell cultures were subjected to the extraction of total RNA using a viral nucleic extraction kit (TIANGEN, China). Nine overlapped fragments covering the whole genome were amplified by RT-PCR as previously described^32^. Recombinant clones were sequenced by Ruibo Life Technologies Corporation (Beijing, China).

Modified live virus (MLV) vaccines based off VR-2332 (Ingelvac PRRS MLV) from Boehringer Ingelheim Vetmedica, Inc. Strains FJZ03 and FJWQ16 have been passaged 3 times in MARC-145 cells for use in the swine study.

### Complete genomic sequence analysis

To identify the evolutionary relationship of FJZ03 and FJWQ16, 38 representative PRRSV strains in GenBank were utilized in sequence alignments and phylogenetic analyses (Table 2). Multiplex sequence alignments were performed using CLUSTAL X (version 1.83). The phylogenetic tree was constructed using the NJ method with MEGA 6.0, the maximum composite likelihood model and the bootstrap confidence value of 1000 replicates^33^. The ORF5 gene sequences were classified according to the global PRRSV classification systems^12^.

**Table 2:** PRRSV strains used in this study.

No.	Name	GenBank accession no.	Origin	No.	Name	GenBank accession no.	Origin
1	FJWQ16	KX758249	China	21	10-10-GX-3	JQ663560	China
2	FJZ03	KP860909	China	22	Em2007	EU262603	China
3	FJYR	KT804696	China	23	FJ1402	KX169191	China
4	11FS11-GD	JX215551	China	24	QYYZ	JQ308798	China
5	TJ	EU860248	China	25	JX143	EU708726	China
6	CH-1a	AY032626	China	26	10-10BJ-2	JX192635	China
7	JA142	AY424271	China	27	10-10FUJ-3	JQ663548	China
8	VR-2332	U87392	China	28	BJ-4	AF331831	China
9	JXA1-R^a^	R FJ548855	China	29	MN184A	DQ176019	U.S.A.
10	JXA1	EF112445	China	30	MN184B	DQ176020	U.S.A.
11	JXA1 P100^b^	KC422725	China	31	HENNAN-XINX	KF611905	China
12	JXA1 P70^b^	FJ548852	China	32	NADC30	JN654459	U.S.A.
13	JXA1 P45^b^	FJ548851	China	33	JL580	KR706343	China
14	JXA1 P15^b^	FJ548855	China	34	CHsx1401	KP861625	China
15	HB-1(sh)/2002	AY150312	China	35	HNjz15	KT945018	China
16	HB-2(sh)/2002	AY262352	China	36	HNyc15	KT945018	China
17	HUN4	EF635006	China	37	HLJA1	KT351739	China
18	NT1^c^	KP179402	China	38	RespPRRS MLV	AF066183	U.S.A
19.	NT2^c^	KP179403	China	39	GZgy15-1	KT358728	China
20	NT3^c^	KP179404	China	40	GD	EU825724	China

^a^Live attenuated vaccine strain of HP-PRRSV JXA1.

^b^Derivatives of *in vitro* passaged HP-PRRSV JXA1 in MARC-145 cells.

^c^Virulence reversion from live attenuated PRRS vaccine JXA1-R.

The recombination event was confirmed using a recombination detection program (RDP v.4.80)^34^ as described in Ramos *et al*.^35^. Potential recombination events were tested by seven different algorithms (RDP, GeneConv, BootScan, MaxChi, Chimera, SiScan, and 3Seq) with Bonferroni correction and the highest acceptable *p* value 0.01. Recombination breakpoints was further analyzed by the Genetic Algorithm for Recombination Detection (GARD) and SimPlot software v.3.5.1^36,37^.

### Animal study design and clinical observation

Twenty-five 21-day-old pigs confirmed to be free of PRRSV, PCV2, PRV, and CSFV were used for this study. Pigs were allowed to acclimate for one week before initiation of the experiments. All pigs randomly divided into 5 groups (5 pigs/group) and were raised separately in different isolation rooms with individual ventilation. The pigs in groups 1 (MLV + FJZ03 challenge group) and 2 (MLV + FJWQ16 challenge group) were vaccinated intramuscularly with a single dose of MLV according to manufacturer’s directions (Ingelvac PRRS^®^ MLV) on day 0. The pigs in group 3 (unvaccinated + FJZ03 challenge group), group 4 (unvaccinated + FJWQ16 challenge group) and group 5 (unvaccinated unchallenged, control) were mock vaccinated with PBS on the same day. Twenty-eight days post immunization (dpi) (0 day post challenge, dpc), groups 1 and 3 challenged with FJZ03 (2 × 10^5^ TCID50/pig, 2 mL), groups 2 and 4 challenged with FJWQ16 (2 × 10^5^ TCID50/pig, 2 mL) by intranasal (1 mL) and intramuscular (1 mL) routes, respectively. The pigs in group 5 received PBS (2 mL) and served as the negative control group.

Rectal temperature was recorded daily from 0 to 14 dpc and blood samples were collected on 0, 4, 7, 11, and 14 dpc for virus titration. The pigs were monitored daily for clinical respiratory disease as previously described^38^, pigs were monitored every day for clinical signs and scored daily for clinical respiratory disease severity using scores ranging from 0 to 6 (0 = normal, 6 = severe). All of the pigs were euthanized on 14 dpc. Lungs were collected from each pig at necropsy and the macroscopic lesions in the lungs were recorded using a scoring system as previously described^38^, the scoring system is based on the approximate volume that the dorsal and ventral surfaces of each lung lobe accounts for the entire lung: the right anterior lobe, right middle lobe, cranial part of the left anterior lobe, and the caudal part of the left anterior lobe were assigned each 10% of the total lung volume, the accessory lobe were assigned 5%, and the right and left caudal lobes each contribute 27.5%. Macroscopic lung lesions were given a score in a blinded fashion by two veterinary pathologists. Lung were collected and fixed in 10% neutral-buffered formalin and routinely processed for histological examination. Microscopic lung lesions were evaluated in a blinded fashion by two veterinary pathologists as described previously^39^.

### Quantification of PRRSV RNA

To attain a relative quantity of viral RNA, TaqMan fluorescent quantitative RT-PCR (RT-qPCR) was performed on all serum samples as described previously^40^. The PCR products of conserved regions within ORF7 for type 2 PRRSV strains (180 base pair) was cloned with the PMD-19T (Takara, Korea) and transformed into DH5a competent cells (TIANGEN, China). Plasmid DNA was extracted by using a plasmid purification kit (TIANGEN, China) and quantified by the Thermo Scientific Varioskan Flash multimode reader. Real-time RT-PCR using Taqman probes was performed to generate a standard curve by known amounts of the serially diluted ORF7-based plasmid standards (10^1^–10^8^ copies/μL). Specific primers for qPCR in this study was performed as described^40^, PRRSV F: 5′-ACAACGGCAAGCAGCAGAA-3′ and PRRSV R: 5′-GAGCGATGATCTTACCCAGCAT-3′ and the PRRSV probe: 5′-FAM-CTGGGYARGATYATCGCCCAGCA-BHQ1-3′. The concentrations in the tested samples were obtained by the Ct values plotted against the known concentration of the ORF7-based plasmid standards.

### Serology

Serum samples were analyzed by ELISA using the PRRS Virus Antibody Test Kit 2XR (IDEXX Laboratories Inc., Westbrook, ME, USA). The serology test was performed by the manufacturer’s instructions. Samples are considered positive for antibodies to PRRSV when sample/positive (S/P) > 0.4.

### Virus neutralization test in serum

Virus neutralization test was performed in the serum as described previously^41^. Briefly, a 100-μl aliquot of each diluted sample was mixed with an equal volume of FJZ03 or FJWQ16 containing 200 TCID50. Each mixture was transferred to MARC-145 monolayers in 96-well plates after incubation at 37 °C for 1 h. The presence of virus-infected cells in each well was determined by IFA. The neutralizing antibody (NA) titers of the sera against the different PRRSV were calculated using the Reed-Muench method^42^. Animals were considered to be protected from viremia when a titer of greater than eight^43^.

### Statistical analysis

The numerical data is expressed as the mean ± S.D. and was analyzed using SPSS 16.0 software. Differences between groups were assessed using ANOVA. Differences were considered statistically significant when *p* < 0.05.
